# Younger age, higher body mass index and lower adiponectin concentration predict higher serum thromboxane B_2_ level in aspirin-treated patients with type 2 diabetes: an observational study

**DOI:** 10.1186/s12933-014-0112-0

**Published:** 2014-08-15

**Authors:** Agnieszka Kaplon-Cieslicka, Marek Postula, Marek Rosiak, Michal Peller, Agnieszka Kondracka, Agnieszka Serafin, Ewa Trzepla, Grzegorz Opolski, Krzysztof J Filipiak

**Affiliations:** 1st Chair and Department of Cardiology, Medical University of Warsaw, Public Central Teaching Hospital in Warsaw, 1a Banacha St., Warsaw, 02-097 Poland; Department of Noninvasive Cardiology and Hypertension, Central Clinical Hospital, the Ministry of the Interior, Warsaw, Poland; Department of Experimental and Clinical Pharmacology, Medical University of Warsaw, Warsaw, Poland; Chair and Department of Internal Diseases and Endocrinology, Medical University of Warsaw, Warsaw, Poland; Medical Centre, Medical University of Warsaw, Warsaw, Poland

**Keywords:** Aspirin, Platelet aggregation, Diabetes mellitus, Insulin resistance, Adipokines

## Abstract

**Background:**

Evidence from the literature suggests diminished acetylsalicylic acid (ASA) treatment efficacy in type 2 diabetes (DM2). High on-aspirin platelet reactivity (HAPR) in DM2 has been linked to poor glycemic and lipid control. However, there are no consistent data on the association between HAPR and insulin resistance or adipose tissue metabolic activity. The aim of this study was to assess the relationship between laboratory response to ASA and metabolic control, insulin resistance and adipokines in DM2.

**Methods:**

A total of 186 DM2 patients treated with oral antidiabetic drugs and receiving 75 mg ASA daily were included in the analysis. Response to ASA was assessed by measuring serum thromboxane B_2_ (TXB_2_) concentration and expressed as quartiles of TXB_2_ level. The achievement of treatment targets in terms of glycemic and lipid control, insulin resistance parameters (including Homeostatic Model Assessment-Insulin Resistance, HOMA-IR, index), and serum concentrations of high-molecular weight (HMW) adiponectin, leptin and resistin, were evaluated in all patients. Univariate and multivariate logistic regression analyses were performed to determine the predictive factors of serum TXB_2_ concentration above the upper quartile and above the median.

**Results:**

Significant trends in age, body mass index (BMI), HOMA-IR, HMW adiponectin concentration, C-reactive protein concentration and the frequency of achieving target triglyceride levels were observed across increasing quartiles of TXB_2_. In a multivariate analysis, only younger age and higher BMI were independent predictors of TXB_2_ concentration above the upper quartile, while younger age and lower HMW adiponectin concentration were predictors of TXB_2_ concentration above the median.

**Conclusions:**

These results suggest that in DM2, the most important predictor of HAPR is younger age. Younger DM2 patients may therefore require total daily ASA doses higher than 75 mg, preferably as a twice-daily regimen, to achieve full therapeutic effect. Higher BMI and lower HMW adiponectin concentration were also associated with less potent ASA effect. This is the first study to demonstrate an association of lower adiponectin concentration with higher serum TXB_2_ level in patients treated with ASA.

## Background

Treatment with acetylsalicylic acid (ASA) has proven effective in the reduction of cardiovascular morbidity and mortality. Type 2 diabetic (DM2) patients are known to be at a very high cardiovascular risk, therefore it could be anticipated that they should potentially benefit the most from ASA treatment [1]. However, results of the Antithrombotic Trialists' Collaboration's meta-analysis of 195 clinical trials with over 135,000 patients, including almost 5000 diabetic patients, suggest that ASA therapy in diabetic patients may be less effective in cardiovascular prevention than in normoglycemic individuals [2]. Concomitant DM2 also increases the risk of high on-aspirin platelet reactivity (HAPR), defined as inadequate inhibition of platelet function, assessed *in vitro* with laboratory tests [3–9]. According to the position paper of the Working Group on Antiplatelet Drugs Resistance, endorsed by the Working Group on Thrombosis of the European Society of Cardiology, laboratory response to ASA treatment should be assessed with aspirin-specific tests evaluating the degree of cyclooxygenase 1 (COX-1) inhibition, either directly, by measuring serum concentration of thromboxane B_2_ (TXB_2_) - a stable metabolite of thromboxane A_2_ (COX-1 product) or indirectly, by assessing platelet aggregation induced by arachidonic acid (COX-1 substrate) [10].

Patients with DM2 exhibit abnormalities of both platelet and plasma hemostasis, and differ from other patients in terms of thrombus structure and kinetics [11]. The pathomechanism of HAPR in patients with DM2 is complex and, so far, not entirely understood [12,13]. It is hypothesized that under hyperglycemic conditions nonenzymatic glycation of COX-1 may competitively inhibit its acetylation by ASA [14]. In addition, nonenzymatic glycation of platelet membrane proteins may result in reduced membrane fluidity, increasing the propensity of platelets to activate [15]. Other possible mechanisms leading to enhanced platelet reactivity under hyperglycemia include osmotic effect of glucose, increased activation of protein kinase C and decreased activation of nitric oxide (NO) - cyclic guanosine monophosphate (cGMP) - cGMP-dependent protein kinase pathway [15–17]. These assumptions are supported by clinical studies demonstrating relationship between HAPR and inadequate glycemic control, as well as *in vitro* studies showing enhanced platelet activation, assessed using flow cytometry, with increasing glucose concentrations despite incubation with ASA [6,7,18–23]. Alongside with poor glycemic control, HAPR in DM2 patients has been linked to high triglyceride, total cholesterol and low-density lipoprotein (LDL) concentrations, as well as low high-density lipoprotein (HDL) concentration [18,20,24]. Hyperglycemia and hyperlipidemia induce oxidative stress, which, in turn, leads to fatty acids peroxidation and, consequently, formation of products that may change physicochemical properties of platelet plasma membrane or - as described for isoprostanoids - act as ligands for platelet membrane receptors, triggering platelet activation and aggregation via alternative pathways [25]. Furthermore, oxidative stress may also augment platelet reactivity indirectly, as it results in endothelial dysfunction, diminished endothelial synthesis of NO and prostacyclin, and attenuation of biological effects of NO [15].

Much as the relationship of HAPR with glycemic and lipid control of DM2 has been well documented, only few small studies regarding the association of platelet reactivity with insulin resistance and adipokine concentrations have been conducted so far [5,26–28]. Among 60 healthy women, a lower insulin sensitivity index was predictive of higher urine 11-dehydro-TXB_2_ concentration and improvement in insulin sensitivity resulted in decreasing urinary 11-dehydro-TXB_2_ excretion [26]. In a group of 55 patients with DM2, higher Homeostatic Model Assessment-Insulin Resistance (HOMA-IR) index was an independent predictor of poor responsiveness to ASA assessed using urine 11-dehydro-TXB_2_ concentration measurement [27]. Increased rates of HAPR observed in obese patients also imply its potential association with insulin resistance [6,21,26]. On the other hand, similar prevalence of poor responsiveness to ASA in patients with type 1 and type 2 diabetes seems to contradict such relationship [29]. Moreover, a negative correlation of serum TXB_2_ concentration with body mass index (BMI) and leptin concentration (which was shown to positively correlate with HOMA-IR) reported by Graziani et al. also question the previously suggested pathogenetic role of insulin resistance in the development of HAPR [28]. Thus, consistent data on the association of HAPR with insulin resistance and adipose tissue metabolic activity are lacking.

In our previous work, we have evaluated the relationship between HAPR assessed with various laboratory methods and genetic factors, clinical variables and routine laboratory parameters in DM2 [30,31]. The aim of this study was to assess the relationship between laboratory response to ASA therapy and insulin resistance, adipose tissue metabolic activity and the achievement of metabolic control in DM2 patients.

## Methods

### Patient population and study design

The study subjects were recruited from a group of patients participating in a prospective, randomized and open-label AVOCADO (Aspirin Vs/Or Clopidogrel in Aspirin-resistant Diabetics inflammation Outcomes) study. The AVOCADO study enrolled 304 consecutive patients aged between 30 and 80 years, presenting to the outpatient clinic of the Central Teaching Hospital of the Medical University of Warsaw between January 2008 and August 2010, with DM2 diagnosed at least 6 months prior to their presentation, who had been taking enteric coated ASA tablets at a dose of 75 mg per day for at least 3 months for primary or secondary cardiovascular prevention. Exclusion criteria included: diet-controlled DM2, treatment with antiplatelet drugs other than ASA, treatment with anticoagulants, chronic treatment with nonsteroidal anti-inflammatory drugs (NSAID) or self-reported use of NSAIDs within the last 10 days, coexisting contraindications to ASA treatment, ASA intolerance, platelet count below 100,000/mm^3^ or above 450,000/mm^3^, hemoglobin concentration below 8 g/dL, a history of bleeding diathesis or other coagulation disorders, malignant disease (current or therapy within the past 5 years), connective tissue disease, end-stage renal disease requiring dialysis, acute coronary syndrome, coronary angioplasty or coronary artery bypass grafting in the last 12 months prior to enrollment into the study, active and clinically overt inflammation, exacerbation of chronic heart failure, acute complications of diabetes and other acute medical conditions requiring unplanned hospitalization in the last 8 weeks prior to the study, and surgical procedures in the previous 8 weeks. A more detailed characteristic of the AVOCADO study population has been published previously [30–32].

The study was conducted in accordance with the current version of the Declaration of Helsinki. The local ethics committee of the Medical University of Warsaw approved both the AVOCADO study protocol and the informed consent form. A written informed consent was obtained from all patients.

For the current analysis, only DM2 patients treated with oral antidiabetic drugs were selected from the AVOCADO study (patients treated with insulin in monotherapy or in combination were excluded).

### Data collection and laboratory tests

Demographic information, medical history, medications, and lifestyle habits were obtained from all patients through a personal interview and a review of medical charts. A thorough physical examination with anthropometric measurements was also conducted.

Venous blood samples were collected in the morning (between 8 a.m. and 9 a.m.) following an overnight fast, 2-3 hours after the last ASA dose (one day before testing all patients were reminded by a telephone call to take their daily ASA at least 2 hours before the appointment). Regular laboratory testing was performed at the laboratory of the Central Teaching Hospital, Medical University of Warsaw using standard techniques and included fasting glycemia, glycated hemoglobin (HbA_1c_), lipid profile, high-sensitivity C-reactive protein (hsCRP), serum creatinine, urea and uric acid concentrations, complete blood cell and platelet counts, and coagulation tests.

Serum (for TXB_2_, insulin, C-peptide and adipokines concentrations measurements) and citrated plasma (for von Willebrand factor activity measurement) had been obtained from venous blood by centrifugation at 1000 g for 15 min at 4°C, and aliquots had been stored at -80°C until patients' enrollment was completed - afterwards the samples were defrosted and appropriate measurements were performed. Concentrations of functional epitope of von Willebrand factor were measured in citrate plasma samples using von Willebrand Factor Activity Kit®, according to the manufacturer’s instructions (American Diagnostica Inc., USA). Von Willebrand factor activity was expressed as percentage (100% corresponds to 1 IU/mL).

#### Measurement of serum thromboxane B_2_

Whole blood for TXB_2_ was allowed to clot at 37°C for 1 hour, after which the serum was separated by centrifugation and stored at -80°C, as described above. Serum TXB_2_ was measured with an enzyme immunoassay kit (Thromboxane B_2_ EIA Kit®), according to the manufacturer’s instructions (Cayman Chemicals, USA). Samples with results outside the standard curve were re-assayed with appropriate dilutions. Given the unequivocal data in the literature, we have decided not to adopt any threshold for serum TXB_2_ concentration that would define HAPR [33–35]. Instead, we regarded serum TXB_2_ concentration as a continous variable, dividing patients into groups consistent with serum TXB_2_ concentration quartiles. Thus, we compared patients with serum TXB_2_ concentrations in increasing quartiles. In order to determine the risk factors for HAPR, we analyzed which variables were predictive of serum TXB_2_ concentration above the upper (third) quartile. Additionally, we analyzed which variables were predictive of serum TXB_2_ concentration above the median (second quartile).

#### Assessment of metabolic control of diabetes

Glycemic and lipid treatment targets were defined in accordance with the American Diabetes Association guidelines as achieving HbA_1c_ below 7%, LDL concentration below 100 mg/dL in patients without overt coronary artery disease and below 70 mg/dL in patients with coronary artery disease, HDL concentration above 50 mg/dL in women and above 40 mg/dL in men, and triglyceride concentration below 150 mg/dL [36].

#### Assessment of insulin resistance

Insulin and C-peptide serum concentrations were measured using Elecsys Insulin Assay® and Elecsys C-Peptide Assay®, respectively, according to the manufacturer’s instructions (Roche Diagnostics, Germany) with electrochemiluminescence, on Elecsys 2010 analyzer (Hitachi High-Technologies Corporation, Japan). Homeostasis Model Assessment-Insulin Resistance (HOMA-IR) index was then calculated based on the following formula: insulin concentration × fasting glycemia × 0.0555/22.5, with insulin concentration expressed in μIU/mL and fasting glycemia expressed in mg/dL.

#### Assessment of adipose tissue metabolic activity

High-molecular weight (HMW) adiponectin, leptin and resistin concentrations were measured in serum using Human High Molecular Weight Adiponectin ELISA® (Millipore Corporation, USA), Human Leptin Quantikine ELISA Kit® (R&D Systems Inc., USA) and Human Resistin Quantikine ELISA Kit® (R&D Systems Inc., USA), respectively.

### Assessment of compliance to ASA therapy

Compliance to ASA therapy was determined based upon patients' statements and serum TXB_2_ concentration measurement. Based on the literature, optimal compliance to ASA therapy was confirmed by serum TXB_2_ concentrations below 7200 pg/mL [9]. Only patients with optimal compliance were included in the final analysis.

### Statistical analysis

Statistical analysis was performed using SAS® software, version 9.2. Normal distribution of variables was assessed using the Shapiro-Wilk test and histograms. When normally distributed, quantitative variables are presented as a mean value ± standard deviation (SD), when non-normally distributed - as a median and interquartile range (IQR). Qualitative variables are presented as absolute and relative frequencies. Statistical significance of trends across increasing quartiles of serum TXB_2_ concentration was assessed: for quantitative variables with Jonckheere-Terpstra test, for qualitative variables - with Cochran-Armitage test for trend. Univariate and multivariate logistic regression analyses were performed to determine the predictive factors of serum TXB_2_ concentration above the upper quartile and of serum TXB_2_ concentration above the median. In a multivariate logistic regression model all factors which were found to be significant in univariate analyses were used. A value of p ≤ 0.05 was considered significant for all tests.

## Results

### Study group selection

Out of 304 patients enrolled in the AVOCADO study, complete clinical data and blood samples were available from 295 patients, including 195 patients treated only with oral antidiabetic agents. Additionally, 9 patients were excluded from the study based on suspected ASA non-compliance (serum TXB_2_ concentration >7200 pg/mL), resulting in a total of 186 patients included in the final analysis.

### Study group characteristics

**Figure 1:**
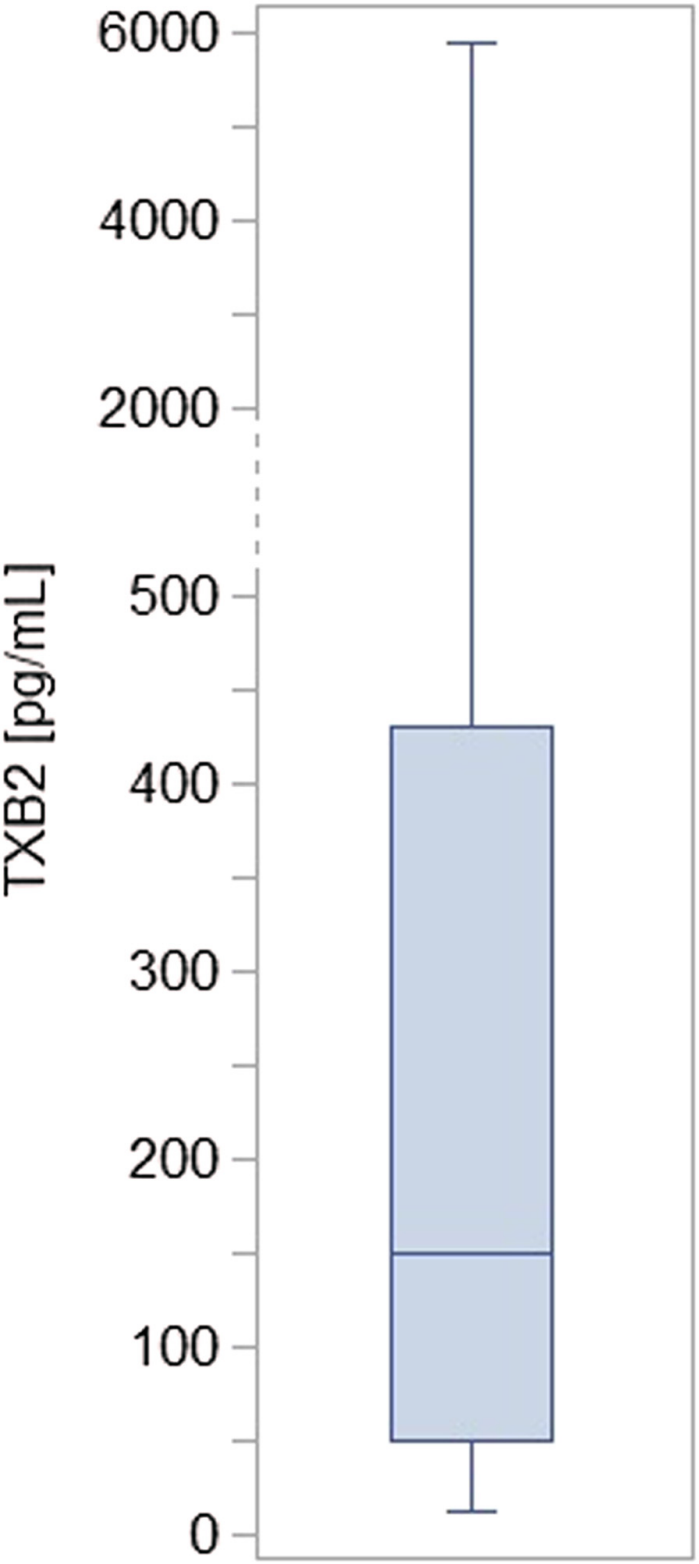
**Distribution of serum thromboxane B**_**2**_ **concentration in the study group.** TXB_2_ - thromboxane B_2_. The box plot represents the median, the interquartile range, and the minimum and maximum value.

Mean age of the study population was 67.7 (±8.7) years. Women constituted 50.5% of the study group. Median serum TXB_2_ concentration was 149.1 pg/mL (minimum: 2.7 pg/mL, maximum: 5812.3 pg/mL). Figure 1 represents the distribution of serum TXB_2_ concentration in the study group. Clinical and laboratory characteristics of the study group in relation to quartiles of serum TXB_2_ concentration are shown in Tables 1 and 2. Patient recruitment began in January 2008, which explains why only two-thirds of the patients received metformin (according to the guidelines valid at the time, metformin had not yet been recommended for the majority of patients with DM2). There were significant trends in the use of metformin and sulfonylurea derivatives associated with serum TXB_2_ concentrations: the frequency of metformin treatment increased and that of sulfonylurea derivatives decreased with increasing quartiles of serum TXB_2_ (Table 1). As the direction of this relationship seemed surprising, given the potential antiplatelet properties of metformin, we compared patients receiving and not receiving metformin, and those receiving and not receiving sulfonylurea derivatives (Table 3) [37]. In comparison to patients not receiving metformin, patients treated with metformin were on average 4 years younger and had a significantly higher BMI (contrary trends were observed for therapy with sulfonylurea derivatives), which could explain the observed relationship of diabetes treatment with increasing quartiles of serum TXB_2_, given a negative association of serum TXB_2_ level with age and its positive association with BMI. Therefore, in order to avoid a methodological bias in determining the risk factors for HAPR, we have decided not to include diabetes treatment in the logistic regression analysis.

**Table 1:** **Clinical characteristics of the study group in relation to platelet reactivity, expressed as quartiles of serum thromboxane B**_**2**_ **concentration**

**Clinical parameters**	**TXB**_**2**_	**TXB**_**2**_	**TXB**_**2**_	**TXB**_**2**_	***p***
	**<50 pg/mL**	**[50-150 pg/mL)**	**[150-450 pg/mL)**	**≥450 pg/mL**	
	***n = 45***	***n = 49***	***n = 47***	***n = 45***	
Age [years]	69.2 (±8.8)	70.0 (±7.4)	66.7 (±8.7)	64.7 (±9.3)	**0.0062**
Female gender [n (%)]	27 (60.0%)	24 (49.0%)	20 (42.6%)	23 (51.1%)	0.32
Duration of diabetes [years]	5 (2-10)	6 (3-10)	6 (3-11)	5 (2-7)	0.36
**Anthropometric parameters**					
BMI [kg/m^2^]	29.1 (±4.3)	29.4 (±4.4)	31.2 (±5.4)	31.8 (±5.3)	**0.005**
SBP [mmHg]	142.6 (±19.4)	141.3 (±18.8)	135.3 (±16.7)	141.9 (±19.3)	0.64
DBP [mmHg]	79.3 (±9.5)	80.9 (±12.1)	79.8 (±10.7)	81.0 (±13.4)	0.56
**Diabetes treatment** [n (%)]					
Metformin	29 (64.4%)	24 (49.0%)	35 (74.5%)	36 (80.0%)	**0.0188**
Sulfonylurea derivatives	30 (66.7%)	40 (81.6%)	35 (74.5%)	22 (48.9%)	**0.0485**
Sulfonylurea derivatives and metformin	15 (33.3%)	18 (36.7%)	25 (53.2%)	14 (31.1%)	0.74
Acarbose	2 (4.4%)	5 (10.2%)	7 (14.9%)	5 (11.1%)	0.22
DPP-4 inhibitors, TZD and glinides	0	0	0	0	-
**Coexisting conditions** [n (%)]					
Hypertension	42 (93.3%)	45 (91.8%)	41 (87.2%)	42 (93.3%)	0.80
Dyslipidaemia	37 (82.2%)	45 (91.8%)	40 (85.1%)	35 (77.8%)	0.39
CAD	19 (42.2%)	31 (63.3%)	22 (46.8%)	26 (57.8%)	0.39
Previous MI	9 (20.0%)	16 (32.7%)	14 (29.8%)	10 (22.2%)	0.91
Chronic HF	12 (26.7%)	21 (42.9%)	15 (31.9%)	18 (40.0%)	0.38
- NYHA class I	1 (2.2%)	6 (12.3%)	2 (4.3%)	5 (11.1%)	0.23
- NYHA class II	8 (17.8%)	9 (18.4%)	10 (21.3%)	11 (24.4%)	
- NYHA class III	3 (6.7%)	6 (12.3%)	3 (6.4%)	2 (4.4%)	
- NYHA class IV	0	0	0	0	
Previous stroke or TIA	5 (11.1%)	5 (10.2%)	4 (8.5%)	2 (4.4%)	0.24
Current smoking	3 (6.7%)	3 (6.1%)	6 (12.8%)	6 (13.3%)	0.16
**Treatment of concomitant diseases** [n (%)]					
β-blocker	33 (73.3%)	29 (59.2%)	32 (68.1%)	32 (71.1%)	0.96
- carvedilol or nebivolol	4 (8.9%)	6 (12.3%)	4 (8.5%)	1 (2.2%)	0.16
ACE-I or ARB	36 (80.0%)	39 (79.6%)	37 (78.7%)	34 (75.6%)	0.60
Aldosterone antagonist	1 (2.2%)	5 (10.2%)	6 (12.8%)	4 (8.9%)	0.23
Other diuretics	25 (55.6%)	21 (42.9%)	26 (55.3%)	21 (46.7%)	0.97
CCB	18 (40.0%)	11 (22.5%)	18 (38.3%)	23 (51.1%)	0.13
Statin	34 (75.6%)	40 (81.6%)	34 (72.3%)	31 (68.9%)	0.27

Relative frequency, standard deviations and interquartile ranges are presented in parentheses.

TXB_2_ - thromboxane B_2_; BMI - body mass index; SBP - systolic blood pressure; DBP - diastolic blood pressure; DPP-4 - dipeptidylpeptidase-4; TZD - thiazolidinediones; CAD - coronary artery disease; MI - myocardial infarction; HF – heart failure; NYHA - New York Heart Association; TIA - transient ischemic attack; ACE-I - angiotensin-converting enzyme inhibitor; ARB - angiotensin receptor blocker; CCB - calcium channel blocker. Bold font indicates *p*-values ≤0.05.

**Table 2:** **Laboratory characteristics of the study group in relation to platelet reactivity, expressed as quartiles of serum thromboxane B**_**2**_ **concentration**

**Laboratory parameters**	**TXB**_**2**_	**TXB**_**2**_	**TXB**_**2**_	**TXB**_**2**_	***p***
	**<50 pg/mL**	**[50-150 pg/mL)**	**[150-450 pg/mL)**	**≥450 pg/mL**	
	***n = 45***	***n = 49***	***n = 47***	***n = 45***	
**Insulin resistance parameters and adipokines**					
HOMA-IR	3.23 (2.29-4.94)	3.54 (2.42-6.07)	4.48 (2.62-7.29)	4.25 (3.16-8.12)	**0.019**
Insulin [μIU/mL]	11.22 (7.66-15.50)	11.54 (8.88-20.17)	14.24 (9.30-21.00)	15.44 (10.01-21.09)	0.076
C-peptide [ng/mL]	3.756 (2.964-4.680)	3.852 (3.096-5.556)	3.948 (3.360-5.316)	4.260 (3.552-4.908)	0.095
HMW adiponectin [μg/mL]	3.86 (2.28-5.20)	3.15 (2.20-5.54)	2.31 (1.55-3.24)	2.41 (1.65-3.40)	**0.002**
Leptin [ng/mL]	14.52 (9.40-22.16)	18.40 (9.02-26.09)	15.54 (9.68-33.02)	19.21 (8.24-37.10)	0.36
Resistin [ng/mL]	6.85 (5.21-9.73)	7.50 (5.91-8.60)	6.55 (5.58-7.97)	6.66 (4.91-8.69)	0.41
**Glycemic control parameters**					
HbA_1c_ [%]	6.2 (6.1-6.6)	6.5 (6.0-7.1)	6.5 (5.9-7.2)	6.6 (6.3-7.6)	**0.018**
HbA_1c_ target achieved	38 (84.4%)	33 (67.4%)	32 (68.1%)	29 (64.4%)	0.051
Fasting glycemia [mg/dL]	116 (102-130)	120 (107-136)	120 (104-147)	126 (108-155)	**0.041**
**Lipid control parameters**					
TC [mg/dL]	159.3 (±46.8)	165.3 (±36.6)	178.1 (±34.0)	164.7 (±34.0)	0.12
LDL cholesterol [mg/dL]	83.3 (±28.4)	87.5 (±31.3)	96.4 (±30.9)	88.9 (±29.4)	0.23
LDL cholesterol target achieved	22 (48.9%)	22 (44.9%)	16 (34.0%)	22 (48.9%)	0.73
HDL cholesterol [mg/dL]	47.8 (±10.1)	51.7 (±17.8)	49.4 (±12.5)	46.4 (±11.2)	0.58
HDL cholesterol target achieved	25 (55.6%)	22 (44.9%)	18 (38.3%)	22 (48.9%)	0.42
TG [mg/dL]	122.0 (±46.0)	125.6 (±59.5)	158.0 (±77.7)	153.2 (±71.5)	**0.010**
TG target achieved	35 (77.8%)	38 (77.6%)	27 (57.5%)	27 (60.0%)	**0.016**
**Other laboratory parameters**					
PLT [10^3^/μL]	214.5 (±64.7)	229.5 (±59.0)	241.4 (±67.1)	224.8 (±44.8)	0.30
MPV [fL]	10.0 (±1.3)	9.9 (±1.2)	9.5 (±1.1)	10.0 (±1.2)	0.65
WBC [10^3^/μL]	6.46 (±1.31)	6.75 (±1.83)	7.26 (±1.53)	6.83 (±1.95)	0.24
RBC [10^6^/μL]	4.73 (±0.43)	4.58 (±0.54)	4.67 (±0.46)	4.66 (±0.40)	0.83
Hemoglobin [g/dL]	14.0 (±1.2)	13.8 (±1.3)	14.1 (±1.2)	14.0 (±1.2)	0.77
Hematocrit [%]	42.2 (±3.5)	41.2 (±3.8)	42.2 (±3.5)	41.6 (±3.3)	0.99
Fibrinogen [mg/dL]	417.2 (±101.3)	443.8 (±106.7)	440.8 (±126.2)	408.3 (±95.5)	0.59
vWF activity (%)	138.3 (96.8-157.9)	141.0 (112.7-185.1)	134.3 (102.3-199.1)	111.8 (89.6-165.1)	0.49
APTT [s]	29.3 (±3.7)	29.2 (±3.2)	28.9 (±4.7)	29.2 (±4.0)	0.82
INR	0.97 (0.93-1.03)	0.96 (0.91-1.00)	0.95 (0.92-0.98)	0.98 (0.92-1.02)	0.79
hsCRP [mg/dL]	1.6 (1.1-3.3)	2.9 (1.5-4.9)	3.2 (2.2-5.7)	2.7 (1.4-4.4)	**0.023**
eGFR [mL/min/1,73m^2^]	74.3 (62.1-90.3)	68.4 (59.3-87.7)	78.1 (67.0-93.2)	77.1 (63.7-91.2)	0.24
Urea [mg/dL]	41.0 (±11.2)	43.3 (±12.7)	37.6 (±11.4)	39.0 (±10.0)	0.092
UA [mg/dL]	5.9 (±1.4)	5.6 (±1.6)	5.6 (±1.4)	5.8 (±1.4)	0.91

Relative frequency, standard deviations and interquartile ranges (IQR) are presented in parentheses.

TXB_2_ - thromboxane B_2_; HOMA-IR - Homeostasis Model Assessment-Insulin Resistance; HMW - high-molecular weight; HbA_1c_ - glycated hemoglobin; TG - triglycerides; HDL - high-density lipoprotein; TC - total cholesterol; LDL - low-density lipoprotein; PLT - platelet count; MPV - mean platelet volume; WBC - white blood cell count; RBC - red blood cell count; vWF - von Willebrand factor; APTT - activated partial thromboplastin time; INR - international normalized ratio; hsCRP - high-sensitivity C-reactive protein; eGFR - estimated glomerular filtration rate; UA - uric acid. Bold font indicates *p*-values ≤0.05.

**Table 3:** **Comparison of patients receiving and not receiving metformin, and patients receiving and not receiving sulfonylurea derivatives**

	**Metformin treatment**		***p***
	**Yes**	**No**	
**Age** [years]	66.2 (±8.4)	70.6 (±8.8)	**0.001**
**BMI** [kg/m^2^]	31.0 (±4.9)	29.1 (±4.9)	**0.014**
	**Sulfonylurea derivatives treatment**		***p***
	**Yes**	**No**	
**Age** [years]	68.4 ± 9.0	66.1 ± 8.0	0.093
**BMI** [kg/m^2^]	29.8 ± 5.0	31.6 ± 4.7	**0.025**

BMI - body mass index. Bold font indicates *p*-values ≤0.05.

### Predictors of serum TXB_2_ concentration above the upper quartile

In univariate analyses, the only predictive factors for serum TXB_2_ level above the upper (third) quartile were younger age (odds ratio, OR [per 5 years] 0.762, 95% confidence interval, CI 0.622-0.932, p = 0.008) and higher BMI (OR [per 5 kg/m^2^] 1.615, 95% CI 1.106-2.360, p = 0.013) - both proved to be independent risk factors in multivariate analysis (Table 4).

**Table 4:** **A predictive model of serum thromboxane B**_**2**_ **concentration above the upper quartile**

**Variables**	**OR**	**95% CI**	***p***
**Age**	**0.785**	**0.637-0.967**	**0.023**
[5 years]			
**BMI**	**1.495**	**1.021-2.190**	**0.039**
[5 kg/m^2^]			

OR - odds ratio; CI - confidence interval; BMI - body mass index. Bold font indicates *p*-values ≤0.05.

### Predictors of serum TXB_2_ concentration above the median

In univariate analyses, predictive factors for serum TXB_2_ level above the median (second quartile) were: younger age (OR [per 5 years] 0.721, 95% CI 0.599-0.868, p < 0.001), higher BMI (OR [per 5 kg/m^2^] 1.706, 95% CI 1.205-2.416, p = 0.003), lower HMW adiponectin concentration (OR [per 1 μg/mL] 0.777, 95% CI 0.665-0.907, p = 0.001), and triglyceride concentration above 150 mg/dL (OR 2.880, 95% CI 1.491-5.562, p = 0.002).

In multivariate analysis only younger age and lower HMW adiponectin concentration (with borderline significance) were risk factors for serum TXB_2_ above the median (Table 5).

**Table 5:** **A predictive model of serum thromboxane B**_**2**_ **concentration above the median**

**Variables**	**OR**	**95% CI**	***p***
**Age**	**0.825**	**0.686-0.993**	**0.042**
[5 years]			
**BMI**	1.376	0.978-1.936	0.067
[5 kg/m^2^]			
**HMW adiponectin**	**0.857**	**0.734-1.000**	**0.050**
[1 μg/mL]			
**TG ≥ 150 mg/dL**	1.651	0.829-3.287	0.154

OR - odds ratio; CI - confidence interval; BMI - body mass index; HMW - high-molecular weight; TG - triglycerides. Bold font indicates *p*-values ≤0.05.

## Discussion

In the studied group of DM2 patients, age proved to be the most important predictive factor of laboratory response to ASA therapy. Some published data suggest an association between HAPR and older age, which could be explained by an increasing number of comorbidities and other coexisting conditions, including risk factors for HAPR [38]. However, our analysis revealed younger age as an independent predictor for HAPR. Similar results have been previously reported by Maree et al. [34]. In a group of 131 stable cardiovascular patients taking 75 mg of enteric-coated ASA daily, HAPR, defined as serum TXB_2_ concentrations above 2200 pg/mL, was found in 44% of patients. In a multivariate analysis, younger age and higher body weight were the only predictive factors of HAPR. In all cases of HAPR *in vitro* incubation of participants’ blood samples with ASA solution resulted in a complete inhibition of platelet aggregation, which implied a pharmacokinetic mechanism of HAPR (i.e. related to diminished bioavailability of ASA) [34].

Lower bioavailability of ASA in younger patients may be attributable to a higher activity of plasma, hepatic and intestinal esterases, which hydrolyze ASA to salicylic acid - a product deprived of permanent antiplatelet activity. Review of published literature confirms that plasma esterase activity, including aspirin esterase, decreases in older people burdened with additional risk factors, especially a coexisting inflammatory state [39–41]. Furthermore, compared with healthy volunteers, higher aspirin esterase activity has been reported in DM2 patients, leading to faster ASA hydrolysis and HAPR [42]. These data suggest that the inverse relationship between serum TXB_2_ concentration and age observed in our study could arise from age-related differences in ASA bioavailability in DM2 patients. It can be hypothesized that in younger DM2 patients a total daily dose of ASA higher than 75 mg may be required to exhibit full antiplatelet effect. Low doses of ASA are a known risk factor for HAPR and increasing ASA doses have been reported to overcome HAPR [6,19,25].

The etiology of HAPR in younger DM2 patients might involve the above described pharmacokinetic mechanism, as well as pharmacodynamic abnormalities. A higher rate of platelet turn-over resulting in a faster recovery of COX-1 activity after ASA administration has been proposed as a potential pharmacodynamic mechanism of HAPR in DM2 after several studies demonstrated increased platelet aggregation at the end of the 24-hour dosing interval, compared to platelet aggregation measured early after ASA ingestion [43–45]. In 100 DM2 patients receiving enteric-coated ASA 100 mg once daily, serum TXB_2_ concentration recovery rate between 12 and 24 hours after ASA intake was measured by Rocca et al. [45]. Younger age, higher BMI and higher mean platelet volume (MPV) were independent predictors of the most rapid increase in TXB_2_ concentration. Positive correlations between TXB_2_ recovery rate and MPV as well as between TXB_2_ recovery rate and the percentage of newly-released, messenger ribonucleic acid (mRNA)-positive platelets were observed, which suggests that the recovery of COX-1 activity during the 24-hour dosing interval largely depends on accelerated platelet turn-over. Most importantly, in a subgroup of 33 patients with the most rapid TXB_2_ recovery on ASA 100 mg o.d., changing ASA regimen to 100 mg b.i.d. resulted in a persistent inhibition of COX-1 activity, while changing to 200 mg o.d. displayed an intermediate pattern of TXB_2_ recovery [45]. Several other platelet function studies conducted in DM2 patients, confirmed the beneficial effect of a twice-daily ASA regimen compared to an equivalent ASA dose given once daily [46–49].

Thus, the accumulated evidence indicates that in DM2 a twice-daily ASA regimen may be favorable compared to merely increasing ASA given as a single daily dose. This approach might be of value especially in younger DM2 patients, as it would counteract both the pharmacokinetic (esterase-activity-dependent) and the pharmacodynamic (related to the increased platelet turn-over) mechanism of HAPR. However, no threshold allowing identification of such "younger" patients can be warranted based on the results of our study, as the observed difference in age between the patients with TXB_2_ concentrations above the upper quartile and the patients with TXB_2_ concentrations below the lower quartile was only 4.5 years. Therefore, clinical significance of this finding should be verified by further studies.

The second independent predictor of serum TXB_2_ concentration above the upper quartile in the studied population was a higher BMI. In a univariate analysis, higher BMI was also significantly predictive of serum TXB_2_ concentration above the median, however, this effect was attenuated in a multivariate analysis. Indices of obesity, including higher BMI and body weight, as well as markers of visceral obesity, such as waist circumference and waist-hip ratio (WHR) have been previously described as risk factors for HAPR [6,21,26,27,34,38]. Platelet hyperaggregability in obese patients may result from enhanced lipid peroxidation due to increased generation of intraplatelet reactive oxygen species and low-grade inflammation, which may be related to coexisting metabolic (e.g. lipid) abnormalities [50–53]. Fatty acid peroxidation may, in turn, induce changes in the physicochemical properties of platelet plasma membrane and formation of products which activate platelets by binding to their surface receptors, thus, triggering HAPR in a pharmacodynamic manner [25]. Nevertheless, the observed association between BMI and HAPR in our study, might have also resulted from reduced bioavailability of an enteric-coated ASA preparation, representing a pharmacokinetic mechanism of HAPR. Recently, in a group of 400 healthy volunteers, Grosser et al. [54] have assessed platelet aggregation and serum TXB_2_ concentration after ingestion of a single oral dose of enteric-coated versus immediate-release ASA. Ingestion of an immediate-release preparation led to a substantial inhibition of platelet aggregation and TXB_2_ generation in all of the patients, while a single dose of enteric-coated preparation was associated with high rates of HAPR - higher if assessed 4 hours after ASA intake compared to the measurement performed 8 hours after ASA ingestion. *Ex vivo* addition of ASA to blood samples of non-responders resulted in amelioration of HAPR, suggesting that in those patients HAPR was related to reduced and delayed absorption of enteric-coated ASA tablets [54]. In our study, we used enteric-coated ASA preparations and collected blood samples relatively early (i.e. 2-3 hours) after ASA ingestion. The extent to which this approach might have influenced the results of our analysis remains uncertain, since in the study by Grosser et al. [54], prolongation of enteric-coated ASA treatment to one week led to overcoming of HAPR in all but one of the 27 patients who were unresponsive to a single dose of the enteric-coated preparation. Still, other studies have demonstrated that compared to immediate-release preparations, enteric-coated ASA tablets are less effective at inhibiting TXB_2_ generation, even if taken chronically [55,56]. Interestingly, in these studies, higher body weight was found to be predictive of HAPR - analogously as in the above cited study by Maree et al. [34,55,56]. It has been hypothesized that due to reduced bioavailability, low doses of enteric-coated ASA may be insufficient in heavier patients with a larger volume of distribution [34]. Thus, a conversion from 75 mg daily of enteric-coated ASA to 75 mg daily of immediate-release ASA has been suggested by Peace et al. [56] for patients over 90 kg, with a further dose increase to 150 mg daily in patients over 120 kg.

Despite significant differences in HOMA-IR across increasing quartiles of serum TXB_2_ concentration, logistic regression analysis did not reveal HOMA-IR as a predictive factor of HAPR in the studied population. This might suggest that the observed relationship of higher TXB_2_ levels with lower HMW adiponectin concentrations is not associated with its insulin-sensitizing properties. Moreover, HMW adiponectin remained borderline significant even after correction for BMI. No association of serum TXB_2_ concentration with neither leptin nor resistin levels would also imply that the relationship of low HMW adiponectin concentrations with increased platelet reactivity is independent from its insulin-sensitizing course of action.

To our knowledge, this is the first study to demonstrate the association of lower HMW adiponectin concentration with higher serum TXB_2_ level in aspirin-treated patients. Under physiological conditions, adiponectin is the hormone most abundantly secreted from adipocytes, with serum concentrations ranging between 3 and 30 μg/mL in healthy individuals [57]. In the blood, it circulates as three different oligomeric complexes, including low-molecular weight (a trimer), medium-molecular weight (a hexamer) and HMW adiponectin - the latter is considered the most biologically active form and constitutes approximately half of adiponectin's plasma amount [57]. In contrast to leptin and resistin, adiponectin levels have been shown to correlate inversely with BMI, the amount of visceral adipose tissue and insulin resistance [58–60]. Low adiponectin concentration is an independent predictive factor of the future development of DM2 [61]. Hypoadiponectinemia has been also associated with an increased risk of metabolic syndrome, hyperlipidemia, nonalcoholic steatohepatitis and polycystic ovary syndrome, as well as hypertension, coronary artery disease and acute coronary syndrome [59,60]. Adiponectin acts via two distinct types of receptors, identified in skeletal muscles (type 1 receptor) and liver (type 2 receptor), as well as on the surface of pancreatic β-cells, endothelial cells, smooth muscle cells, cardiomyocytes, monocytes, macrophages and platelets. It improves insulin sensitivity by increasing fatty acid β-oxidation and glucose utilization, as well as decreasing lipogenesis and gluconeogenesis in the liver. Unlike leptin and resistin, adiponectin has also been shown to exert anti-inflammatory and antiatherogenic effects. Its protective influence on the endothelium results largely from the enhancement of NO generation and attenuation of oxidative stress [57,59,60]. Recently, Okada-Iwabu et al. [62] have discovered an orally active, synthetic adiponectin receptor agonist (AdipoRon), which was found to ameliorate insulin resistance, glucose intolerance and diabetes in experimental rodent models, offering a new promising therapeutic approach to DM2 prevention and treatment.

Although antiplatelet properties of adiponectin have been already described before, the existing evidence stems mostly from *in vitro* studies and the exact mechanism of action remains unknown. Data from the literature imply a possibility of both, a direct and an indirect effect of adiponectin on platelet function. It has been demonstrated that human and animal platelets contain mRNA for adiponectin receptors and that in healthy volunteers, as well as in patients with the metabolic syndrome, platelets express both types of adiponectin receptors on their surface [63,64]. In comparison to wild-type mice, adiponectin knockout mice showed enhanced platelet aggregation induced by adenosine diphosphate (ADP) and collagen, and accelerated thrombus formation on carotid arterial injury [63]. *In vitro* incubation of platelets with adiponectin decreased spontaneous, epinephrine- and ADP-induced platelet aggregation in several, although not in all studies [64–66]. Furthermore, adiponectin could influence platelet reactivity also in an indirect manner, by counteracting the deleterious effects of perioxide generation and by increasing the activity of the endothelial NO synthase (eNOS), NO generation, endothelial COX-2 expression and prostacyclin production [67–69]. Considerably less evidence is available from the clinical studies. In a group of 30 healthy volunteers and 30 patients with the metabolic syndrome, adiponectin concentration correlated negatively with plasma soluble P-selectin and soluble CD40 ligand (CD40L) levels, as well as with epinephrine- and ADP-induced platelet aggregation [64]. In 277 patients with DM2 low concentration of adiponectin was an independent predictive factor for the formation of leukocyte-platelet aggregates [70]. So far, only one study has investigated the relationship between adiponectin level and laboratory response to ASA. Following the incubation of blood samples with ASA solution, Takahashi et al. assessed platelet reactivity with Platelet Function Analyzer-100® (PFA-100®) in 168 men without known cardiovascular disease [5]. Poor responsiveness to ASA was defined as collagen-epinephrine closure time (CEPI-CT) below 250 seconds. The subgroup of 40 poor responders was characterized by a higher prevalence of diabetes and, surprisingly, by a higher adiponectin concentration (8.8 ± 4.1 μg/mL versus 7.3 ± 2.9 μg/mL, p = 0.010). These results, however, were not verified with a multivariate analysis [5].

In the present study, we observed associations of serum TXB_2_ concentration with fasting glycemia, HbA_1c_ proportion and triglyceride concentration. Nevertheless, the results of logistic regression analysis did not support the existence of a relationship between achieving treatment targets [i.e. HbA_1c_ below 7% and triglyceride level below 150 mg/dL] and serum TXB_2_ levels. There was no association of serum TXB_2_ concentration with total, LDL or HDL cholesterol. This is not in concordance with the data from the previous studies, which have reported a significant relationship between HAPR and the indices of metabolic control of diabetes [6,7,18–22,24]. However, a more thorough analysis of the available literature reveals that studies which showed such relationship mostly included patients with poor or suboptimal diabetes control and, in some cases, patients with diabetes and healthy controls. Such characteristics of a studied population would result in a broader spectrum of values of the analyzed parameters, which could facilitate a demonstration of significance between the observed differences. Furthermore, the higher glucose, triglyceride and cholesterol concentrations, the higher the probability of development of platelet function disorders severe enough to be reflected by laboratory tests. On the other hand, patients included in our analysis comprised a more homogeneous group and were characterized by a relatively good metabolic control, with median HbA_1c_ of 6.5% [in comparison to 7.3% in a study by Cohen et al., and 9.4% in a study by Watala et al.], mean total cholesterol of 166.9 mg/dL (in comparison to 207 mg/dL in a study by Watala et al.), mean LDL cholesterol of 89.1 mg/dL (in comparison to 135.4 mg/dL in a study by Hovens et al.), mean HDL cholesterol of 48.9 mg/dL (in comparison to 43 mg/dL in a study by Watala et al.), and mean triglyceride concentration of 139.6 mg/dL (in comparison to 159.4 mg/dL in a study by Hovens et al.) [18,20,21]. These differences may at least partly account for the lack of relationship between platelet reactivity and metabolic control in our study. Another explanation for the lack of significant association between serum TXB_2_ concentration and glycemic control may derive from the findings of Russo et al., who proved that incubation of platelets with hyperosmolar glucose solutions prevented ASA-induced activation of the NO - cGMP - cGMP-dependent protein kinase pathway and, consequently, reduced the ability of ASA to inhibit platelet responses to agonists but did not modify ASA-dependent inhibition of TXB_2_ synthesis [17].

### Limitations of the study

In our study, we evaluated on-aspirin platelet reactivity by measuring serum TXB_2_ concentration. We did not use light transmission aggregometry (LTA), which is traditionally considered “the gold standard” for platelet function assessment. However, as mentioned above, measurement of serum TXB_2_ level is one of the methods recommended by the Working Group on Antiplatelet Drugs Resistance [10]. Moreover, LTA has some important limitations, such as lack of standardization, relatively little reproducibility of results, little correlation with other methods of platelet function assessment and the use of platelet-rich plasma which poorly reflects the *in vivo* conditions of platelet activation. Most importantly, measurement of concentration of serum TXB_2_ (a metabolite of TXA_2_ which is the direct product of COX-1) is the most specific method for the assessment of laboratory response to ASA therapy.

Given the fact that in DM2 patients treated with a single daily dose of ASA, accelerated COX-1 activity recovery has been previously reported (as discussed above), we might have obtained a wider spectrum of serum TXB_2_ concentrations if we had collected blood samples 24 hours instead of 2-3 hours after ASA ingestion. This might have increased the statistical power of our analysis.

Characteristics of the study group did not include some potentially relevant clinical data, such as prevalence of microvascular complications (retinopathy, microalbuminuria), some macrovascular complications (peripheral artery disease, asymptomatic carotid stenosis) and neuropathic or mixed diabetes complications, i.e. diabetic foot syndrome - we did not have these data in the AVOCADO study. Also, data on the use of proton-pump inhibitors were not available.

Another limitation of this study is a relatively small number of patients.

## Conclusions

In the studied group of DM2 patients, the most important independent predictor of HAPR was younger age. Younger patients with DM2 may therefore require total daily ASA doses higher than 75 mg, preferably as a twice-daily regimen, to achieve full therapeutic effect. Higher BMI and lower HMW adiponectin concentration were also associated with less potent ASA effect. This is the first study to demonstrate an association of lower adiponectin concentration with higher serum TXB_2_ level in patients treated with ASA.

## Abbreviations

ASA: Acetylsalicylic acid
BMI: Body mass index
CI: Confidence interval
COX: Cyclooxygenase
DM2: Type 2 diabetes mellitus
HAPR: High on-aspirin platelet reactivity
HMW: High-molecular weight
HOMA-IR: Homeostatic Model Assessment-Insulin Resistance
OR: Odds ratio
TXB_2_: Thromboxane B_2_
